# Beyond Numbers: CKD-EPI Versus MDRD in Primary Care—Differences in Chronic Kidney Disease Stage Classification in 117,055 Patients

**DOI:** 10.3390/jcm15052040

**Published:** 2026-03-07

**Authors:** Nuno Capela, Tiago Taveira-Gomes, Cristina Gavina

**Affiliations:** 1 Family Health Unit, Unidade de Saúde Familiar Serpa Pinto, ULS Santo António, 4250-384 Porto, Portugal; 2 Department of Community Medicine, Information and Decision in Health, Faculty of Medicine, University of Porto, 4200-319 Porto, Portugal; tiago.taveira@med.up.pt; 3 MTG Research and Development Lab, 4200-604 Porto, Portugal; 4 Cardiology Department, Pedro Hispano Hospital, Senhora da Hora, 4464-513 Matosinhos, Portugal; cristinagavina@gmail.com

**Keywords:** chronic kidney disease, glomerular filtration rate, MDRD equation, CKD-EPI equation, primary health care

## Abstract

**Background/Objectives**: Chronic kidney disease (CKD) is a global public health concern, posing significant diagnostic and management challenges in primary care. Estimated glomerular filtration rate (eGFR) is central to CKD staging, yet different estimating equations may yield substantially different stage classifications when applied to the same population. This study aims to compare the eGFR-based CKD stage classification and stage distribution obtained using the Chronic Kidney Disease: Epidemiology Collaboration (CKD-EPI) and Modification of Diet in Renal Disease (MDRD) equations in a large primary care cohort, and to explore the implications of these classification differences for routine use in primary healthcare (PHC). **Methods**: A cross-sectional analysis was conducted using standardized electronic health records from 117,055 PHC patients in the Matosinhos Health Unit, Portugal, spanning 22 years (2000–2022). CKD staging followed KDIGO guidelines and focused on stages G3–G5, based on the most recent available serum creatinine value. CKD-EPI and MDRD equations were compared overall and across age strata, BMI categories, albuminuria categories (when available), and major comorbidity subgroups, including heart failure, diabetes, and hypertension. **Results**: Using CKD-EPI, a higher proportion of individuals were classified as CKD stages G3–G5 (9042; 7.73%) compared with MDRD (7686; 6.57%). Classification differences were most pronounced in advanced stages (relative increase with CKD-EPI: G3b +29.4%, G4 +23.6% and G5 +34.4%). Among individuals aged ≥80 years, equation-related classification differences were particularly marked in advanced stages (G5). Similarly, CKD-EPI was associated with higher CKD stage classification rates in high-risk subgroups, including patients with heart failure. **Conclusions**: Compared with MDRD, CKD-EPI yields a higher proportion of individuals classified into CKD stages, particularly advanced stages and among older adults and high-risk comorbidity subgroups. These findings highlight the substantial impact of equation choice on CKD stage classification in primary care and support the use of CKD-EPI for standardized eGFR reporting, while emphasizing that observed differences reflect classification rather than confirmed CKD diagnosis.

## 1. Introduction

Chronic kidney disease (CKD) affects 10–13% of the global population [1], with prevalence increasing due to aging populations and rising rates of diabetes and hypertension [2,3]. CKD progression leads to mortality and end-stage kidney disease, with costs rising by a factor of four between early and late stages [4]. Early identification of reduced kidney function through glomerular filtration rate (GFR) estimation supports timely intervention and optimization of healthcare resource allocation [5]. Both reduced GFR and its rate of decline independently predict mortality and kidney failure [6]. 

Clinical GFR estimation relies on two equations: Modification of Diet in Renal Disease (MDRD) [7] and CKD Epidemiology Collaboration (CKD-EPI) [8]. These equations combine serum creatinine concentration with demographic factors to estimate kidney function [9,10,11,12,13]. 

Research in GFR estimation has progressed through distinct phases, each addressing limitations of previous approaches. The MDRD equation introduced standardization to GFR estimation, using data from 1628 CKD patients with measured GFR values below 60 mL/min/1.73 m^2^ [7]. Validation studies identified systematic bias in populations with preserved kidney function, limiting its utility for accurate GFR estimation and early CKD stage classification [14]. The CKD-EPI equation addressed these limitations through its development in a broader population (*n* = 8254), including both CKD patients and individuals with normal kidney function [8]. Initial validation demonstrated reduced bias across GFR ranges, particularly above 60 mL/min/1.73 m^2^ [15,16]. Meta-analyses have quantified systematic differences between equations across populations. Meta-analyses have reported improved risk prediction for mortality and end-stage kidney disease with CKD-EPI compared to MDRD [9].

Although a race-free version of CKD-EPI was later introduced [17], the 2009 equation remains widely adopted due to validation and comparability considerations in many regions, including Portugal. 

Equation performance, indeed, varies systematically with population characteristics [18,19,20,21,22]. Primary care studies reveal divergent impacts on clinical decision-making, with CKD-EPI often reclassifying patients to different CKD stages. Age-stratified analyses consistently show increasing divergence between equations in elderly populations, with CKD-EPI often classifying a higher proportion of older individuals into CKD stages [23,24]. At the healthcare system level, equation choice has been shown to influence resource utilization, including referral patterns to nephrology services [25,26]. 

Despite these findings, important gaps remain regarding the application of GFR estimating equations in primary care. Existing studies often lack integrated analyses combining demographic, clinical, and system-level factors, and implementation guidance for resource planning and workflow adaptation remains limited [27,28].

To address these gaps, which have direct implications for clinical decision-making and specialist referral pathways [29,30,31], the present study conducts a comprehensive analysis of 117,055 primary care patients from the Matosinhos Health Unit, Portugal.

We aimed to quantify how the CKD-EPI (2009) and MDRD equations differ in eGFR-based CKD stage classification in a large primary care cohort, focusing on: differences in the proportion of individuals classified as CKD stages G3–G5 and the distribution across G3a–G5; age-stratified differences, particularly in older adults; and differences across clinically relevant subgroups, including BMI categories, albuminuria categories (when available) and major comorbidities (e.g., diabetes, hypertension and heart failure).

## 2. Materials and Methods

### 2.1. Study Design

This cross-sectional study analyzed electronic health records from the Local Health Unit of Matosinhos (Unidade Local de Saúde de Matosinhos; ULSM) Health Unit, Portugal, covering the period between January 2000 and May 2022. The health network includes 14 primary care units and Pedro Hispano Hospital, serving a defined population through centralized laboratory services. 

### 2.2. Population and Data Collection

The study included 117,055 adults (≥18 years) who had contact with the healthcare system within three years before 31 May 2022, representing approximately 82% of the regional adult population (42.47% male). 

Electronic health records provided demographic and anthropometric data, laboratory results (including standardized metabolic, lipid and cardiac panels), and comorbidity information using standardized coding systems (ICD-9/10 and ICPC-2). Ethnicity is not systematically recorded in Portuguese primary healthcare electronic health records and was therefore not available for analysis in the present study.

Key clinical definitions included hypertension (defined by a systolic blood pressure (SBP) ≥ 140 mmHg or diastolic blood pressure (DBP) ≥ 90 mmHg or specific codes), type 2 diabetes mellitus (defined according to internationally accepted diagnostic criteria, including HbA1c ≥ 6.5%, random plasma glucose ≥ 200 mg/dL or specific diagnosis codes, in line with American Diabetes Association guidelines [32]) and heart failure (HF).

Heart failure (HF) was identified using diagnostic codes in combination with left ventricular ejection fraction (LVEF) values extracted from echocardiography reports and B-type natriuretic peptide (BNP) or N-terminal pro-B-type natriuretic peptide (NT-proBNP) levels recorded in the electronic health records.

HF phenotypes were classified according to 2021 European Society of Cardiology guidelines [33] as follows: HF with preserved ejection fraction (HFpEF): LVEF ≥ 50%; HF with mildly reduced ejection fraction (HFmrEF): LVEF 41–49%; HF with reduced ejection fraction (HFrEF): LVEF ≤ 40%.

For HF identification, LVEF criteria were combined with elevated natriuretic peptide concentrations defined as BNP ≥ 100 pg/mL or NT-proBNP ≥ 200 pg/mL. In patients with documented atrial fibrillation, higher natriuretic peptide thresholds were applied (BNP ≥ 125 pg/mL or NT-proBNP ≥ 600 pg/mL) to account for potential biomarker elevation related to arrhythmia. Higher natriuretic peptide thresholds were selected to increase specificity in the context of retrospective electronic health record data.

The population exhibited primary care comorbidity patterns including hypertension (47.58%), diabetes (25.77%) and heart failure (3.03%), with 36.54% classified as overweight and 22.78% as obese. Laboratory measurements followed standardized quality control procedures with participation in external quality assessment programs. Urinary albumin-to-creatinine ratio (UACR) measurements were performed according to standardized protocols; however, UACR data were not available for all individuals. Analyses involving albuminuria were therefore restricted to participants with available UACR measurements.

### 2.3. GFR Estimation Equations

Serum creatinine concentration measurements were performed within a centralized laboratory network and by the same method, ensuring analytical standardization across facilities. This standardized approach was maintained throughout the period, minimizing inter-assay variability over time and allowing consistent application of creatinine-based estimating equations.

Estimated glomerular filtration rate (eGFR) was calculated using both the MDRD equation and the CKD-EPI equation, as follows:

- MDRD equation [8]:

eGFR MDRD = 175 × (SCr)^−1.154^ × age^−0.203^ × 0.742 [if female]

where SCr is serum creatinine concentration (mg/dL), age is expressed in years, and eGFR is expressed in mL/min/1.73 m^2^

- CKD-EPI equation [8]:

eGFR CKD-EPI = 141 × min (SCr/κ, 1)^α^ × max (SCr/κ, 1)^−1.209^ × 0.993^age^ × 1.018 [if female]

where κ = 0.7 (female) or 0.9 (male), and α = −0.329 (female) or −0.411 (male). SCr is serum creatinine concentration (mg/dL) and age is expressed in years.

### 2.4. Statistical Analysis and CKD Staging

CKD staging followed the Kidney Disease: Improving Global Outcomes (KDIGO) guidelines [31]. For each individual, only the most recent available serum creatinine measurement within the study period was used to calculate eGFR and determine CKD stage classification, focusing on stages G3–G5. No attempt was made to confirm chronicity through repeated measurements over a minimum three-month interval. Consequently, transient reductions in kidney function, including possible acute kidney injury (AKI), cannot be fully excluded. The present analysis, therefore, reflects eGFR-based CKD stage classification rather than confirmed chronic kidney disease diagnosis (Table 1).

The primary analysis compared eGFR-based CKD stages classification between CKD-EPI and MDRD, including absolute differences (CKD-EPI minus MDRD percentage) and relative differences ((CKD-EPI minus MDRD)/MDRD × 100%). Age stratification was performed by decade (from 18 to 29 years through ≥80 years). BMI categories followed World Health Organization criteria: underweight (<18.5 kg/m^2^), normal (18.5–24.9 kg/m^2^), overweight (25.0–29.9 kg/m^2^) and obese (≥30.0 kg/m^2^). Quality control measures included evaluation of missing data, outlier detection and verification of measurement consistency.

### 2.5. Ethical Approval

The study received ethics committee approval from the Ethical Committee and Data Protection Officer of ULSM [approval 34/CE/JAS of 23 April 2020 (original) and 64/CE/JAS of 10 July 2020 (addenda)]. 

Patient anonymity was strictly maintained through data coding, ensuring compliance with data protection regulations.

### 2.6. Use of Generative AI Tools

During manuscript preparation, generative AI tools (Microsoft Copilot, based on GPT-4 architecture and Google Gemini, version 1.5) were used exclusively for language editing, grammar correction and rephrasing of selected text segments. These tools were not used for data analysis, statistical computation, the generation of results or scientific interpretation. All scientific content, analyses and conclusions were independently developed, reviewed and validated by the authors.

## 3. Results

A total of 117,055 adults were included (42.47% male; 56.8% aged ≥50 years). Baseline characteristics are summarized in Table 2 and Table 3. The primary results focus on differences in eGFR-based CKD stage classification between CKD-EPI and MDRD, overall and across age, BMI, albuminuria (when available) and comorbidity subgroups.

### 3.1. CKD Stage Classification Differences (G3–G5)

Using CKD-EPI, a total of 9042 individuals (7.73%) were classified as CKD stages G3–G5, compared with 7686 individuals (6.57%) using the MDRD equation (Table 4). Differences in stage classification increased with advanced CKD stage severity.

### 3.2. Age-Stratified Classification Differences

**Figure 1:**
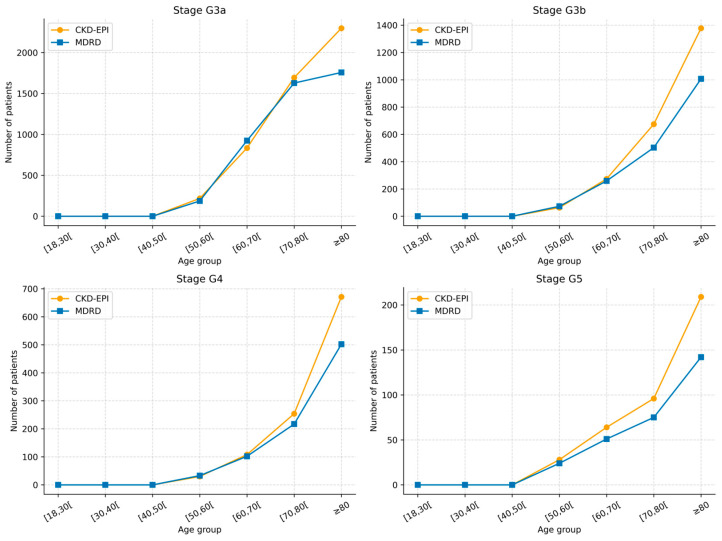
CKD stage classification by estimating equation across age groups.

In individuals aged over 50 years, CKD-EPI consistently resulted in a higher proportion of individuals classified into CKD stages compared with MDRD across all stages (Table 5 and Figure 1).

### 3.3. Body Mass Index (BMI)-Stratified Classification Differences

**Figure 2:**
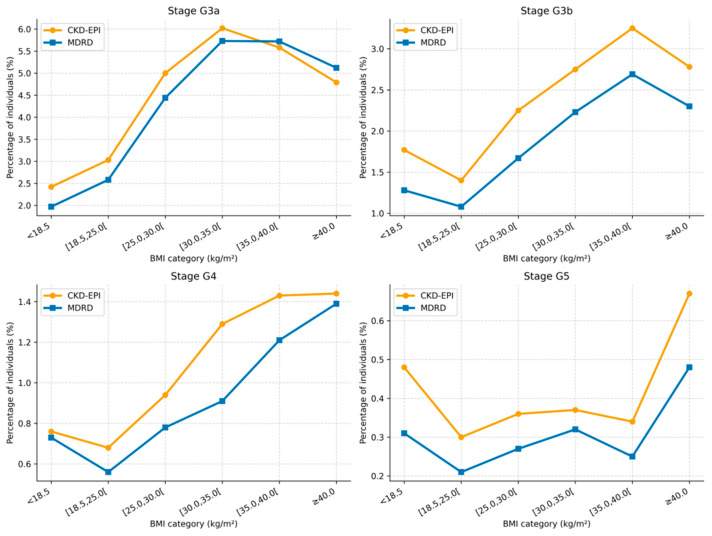
CKD stage classification by estimating equation across BMI categories (percentage).

**Figure 3:**
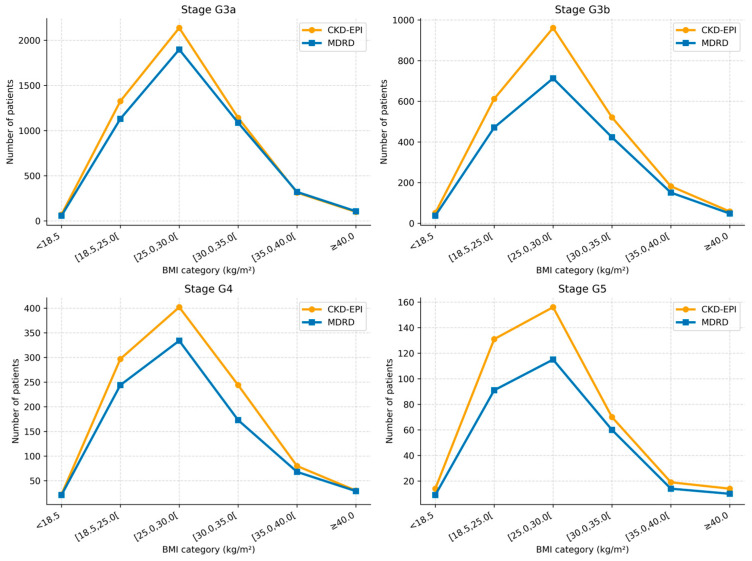
CKD stage classification by estimating equation across BMI categories (number of patients).

BMI stratification revealed consistent differences in CKD stage classification between CKD-EPI and MDRD, with CKD-EPI classifying a higher proportion of individuals as CKD across BMI categories (Table 6 and Table 7, Figure 2 and Figure 3).

### 3.4. Comorbidity Impact

**Figure 4:**
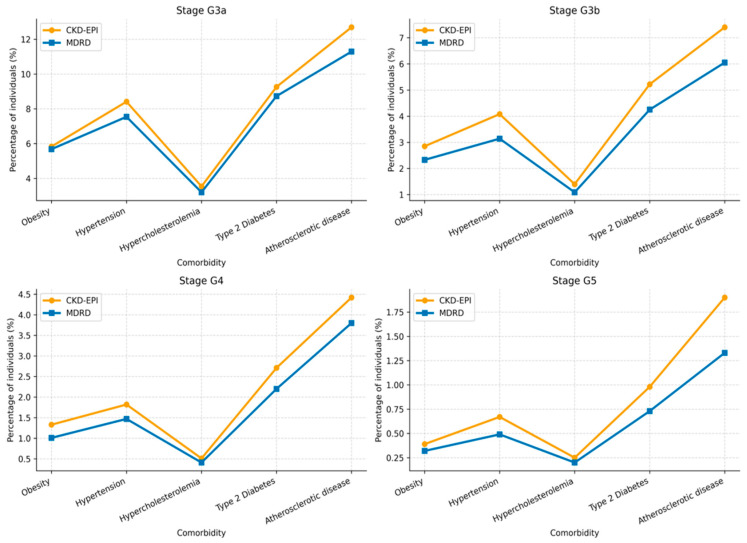
CKD stage classification by estimating equation, stratified by comorbidity: Obesity, hypertension, hypercholesterolemia, type 2 diabetes and atherosclerotic disease.

Differences in CKD stage classification between CKD-EPI and MDRD across major comorbidity subgroups are summarized in Table 8 and Figure 4.

**Figure 5:**
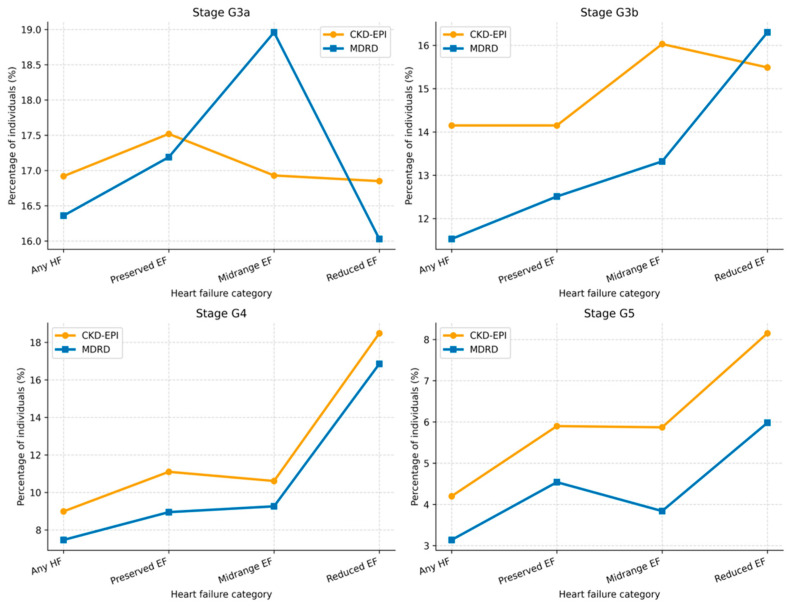
CKD stage classification by estimating equation in patients with heart failure.

Heart failure patients (*n* = 3547) showed increased CKD stage classification with CKD-EPI (Table 9 and Figure 5).

### 3.5. Albuminuria Analysis

UACR data were available in 44,133 individuals (37.7% of the total population), while 72,922 (62.3%) had no recorded albuminuria measurement.

**Figure 6:**
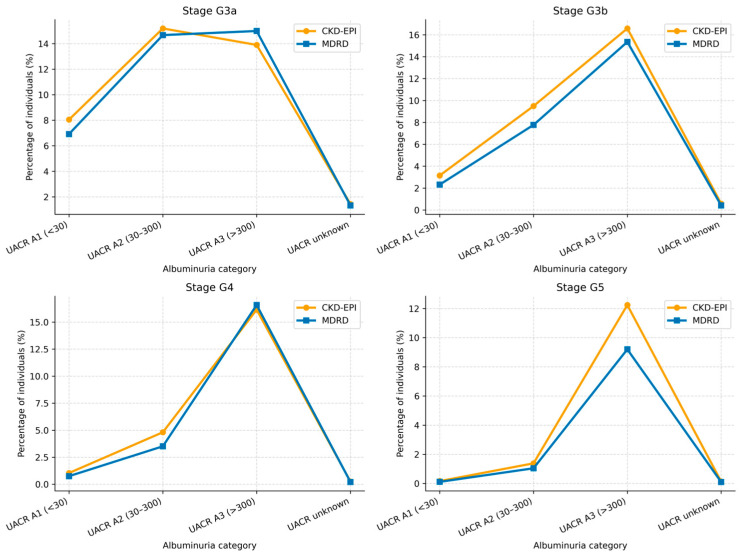
CKD stage classification by eGFR stage and albuminuria category.

**Figure 7:**
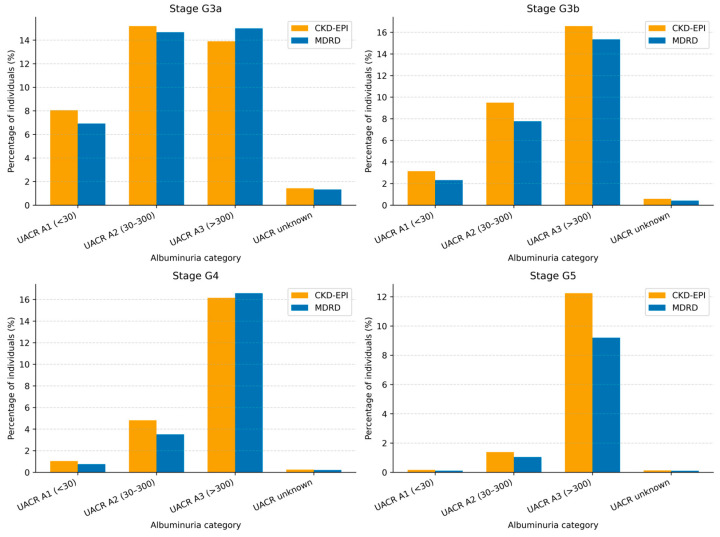
CKD stage classification by eGFR stage and albuminuria category.

**Figure 8:**
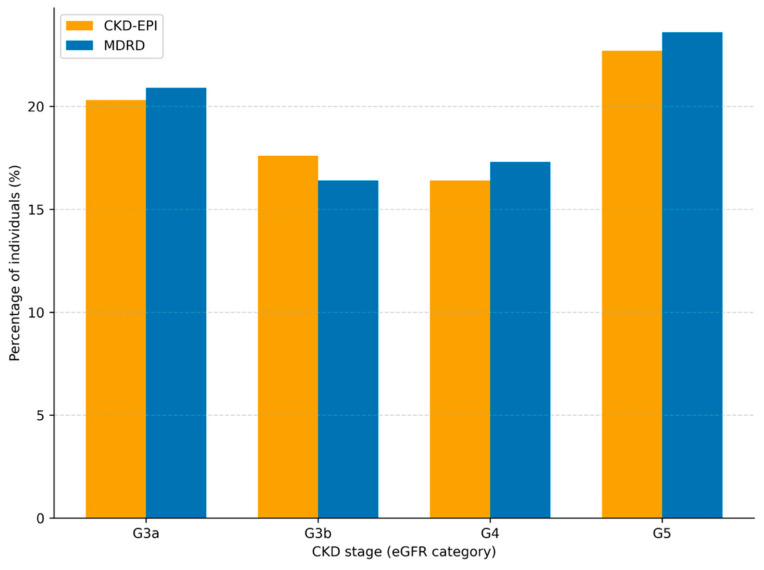
CKD stage classification by eGFR stage and estimating equation among patients without albuminuria.

Albuminuria-stratified analysis demonstrated differences in eGFR-based CKD stage classification across categories (Table 10 and Table 11, Figure 6, Figure 7 and Figure 8). In albuminuria categories A1 and A2, CKD-EPI classified a higher number of individuals across CKD stages compared with MDRD. In category A3, differences between the two equations were smaller, with variation in stage classification across CKD stages.

Among patients with CKD in stages G3a to G5, between 16.4% and 23.6% had no record of UACR.

## 4. Discussion

It is important to emphasize that this study evaluates differences in eGFR-based classification rather than clinical outcomes and therefore does not allow conclusions regarding prognostic superiority or clinical effectiveness of one equation over the other.

In this real-world study, conducted with a representative sample of the adult population in primary healthcare (PHC), the CKD-EPI equation classified a higher proportion of individuals into CKD stages G3–G5 compared with the MDRD equation. This corresponded to an absolute difference of 1.16 percentage points (7.73% vs. 6.57%) and a relative increase of 17.7% in G3–G5 classification when CKD-EPI was used. These differences in eGFR-based stage classification were observed across all estimated glomerular filtration rate (eGFR) intervals, with greater divergence in more advanced stages.

The literature corroborates these findings. Matsushita et al. documented that CKD-EPI estimates eGFR with greater accuracy than MDRD, reclassifying 34.7% of participants from the 45–59 mL/min (MDRD) range to the 60–89 mL/min (CKD-EPI) range, and generally classifying fewer individuals with CKD, highlighting its higher specificity in populations with near-normal kidney function [9]. Brañez-Condorena et al., in a systematic review, observed that, while both equations tend to overestimate eGFR in individuals with CKD and underestimate it in individuals without CKD, CKD-EPI showed a slight advantage in precision and sensitivity, especially for eGFR ≥60 mL/min/1.73 m^2^ [34]. The meta-analysis by McFadden et al. further supports that CKD-EPI presents less bias and greater accuracy, particularly for eGFR values above 60 mL/min/1.73 m^2^, although differences were not significant for lower values [18]. These authors also highlight the scarcity of studies based on community populations, reinforcing the relevance of analysis performed in real-world PHC settings.

Thus, these findings underscore the importance of continuously evaluating GFR estimating equations across different population contexts. The differential behavior of estimating equations according to population characteristics highlights the role of CKD-EPI primarily as a classification and risk stratification tool, rather than as a definitive diagnostic instrument. Our results are consistent with population-based studies, such as the one conducted in Oxfordshire (United Kingdom), which demonstrate an increased identification of advanced CKD by CKD-EPI, particularly in older adults and patients with multimorbidity [35]. This pattern is especially relevant in PHC settings, where multimorbidity and care burden are prevalent and where equation choice can substantially influence clinical workflows and resource planning.

### 4.1. Age and Body Mass Index (BMI)

Differences in CKD stage classification between CKD-EPI and MDRD became more pronounced with increasing age and CKD stage severity. In this study, CKD-EPI consistently classified a higher proportion of older adults into CKD stages—particularly in advanced stages—compared with MDRD, with larger relative differences observed in the oldest age groups. This age-dependent pattern is consistent with previous evidence showing that, in young and middle-aged adults, CKD-EPI tends to yield slightly higher eGFR estimates than MDRD, thereby reducing classification into CKD stages such as G3a for individuals with eGFR values near 60 mL/min/1.73 m^2^ [8,23]. With advancing age, however, this difference diminishes and may invert, resulting in higher CKD stage classification rates when CKD-EPI is used, as observed in the present study. In line with these findings, analyses from the SPRINT trial reported more frequent reclassification into advanced CKD stages among participants aged over 75 years when eGFR was calculated using CKD-EPI rather than MDRD [36].

These age-related differences likely reflect structural characteristics of the estimating equations. The MDRD equation was developed in a CKD population and excluded individuals over 70 years of age, whereas CKD-EPI was derived from a more heterogeneous cohort, albeit with limited representation of very elderly individuals [8]. Both equations may overestimate true glomerular filtration rate (GFR) in older adults due to age-related reductions in muscle mass and creatinine production [11]. Although validation studies have reported lower bias for CKD-EPI at higher eGFR values, its age coefficient may lead to lower eGFR estimates for the same creatinine concentration at advanced ages, thereby increasing CKD stage classification in older populations [8,16]. 

A similar pattern was observed across BMI categories. CKD-EPI consistently resulted in higher CKD stage classification rates compared with MDRD, with stage G3a being the most prevalent across all BMI groups. Differences were less pronounced at extreme BMI values (≥35 kg/m^2^), where absolute differences in classification between equations were smaller. As with age, these findings likely reflect the influence of body composition on creatinine generation, as increased BMI does not necessarily correspond to proportional increases in muscle mass. In individuals with obesity or sarcopenia, creatinine-based equations may therefore be particularly prone to classification differences [21].

Evidence from the literature supports this interpretation. Meta-analyses suggest that alternative equations such as BIS1 and FAS may capture physiological kidney functional decline associated with aging more appropriately [24], while obesity has been consistently associated with increased risk of reduced eGFR and albuminuria [37,38]. Together, these findings highlight the challenge of distinguishing pathological CKD from physiological kidney aging or body composition-related effects. They also support cautious interpretation of eGFR values in older adults and individuals with extreme BMI, as well as the systematic integration of complementary markers, such as urinary albumin–creatinine ratio (UACR) or cystatin C, to refine kidney risk stratification in these populations [16]. 

### 4.2. Comorbidities

Across major cardiometabolic comorbidities, including hypertension, dyslipidemia, type 2 diabetes mellitus, atherosclerotic disease and heart failure, the CKD-EPI equation consistently classified a higher proportion of individuals into CKD stages G3a–G5 compared with MDRD, with consistent relative increases across subgroups. This pattern was observed across all analyzed subgroups, highlighting the substantial impact of equation choice on CKD stage classification in populations with high cardiovascular and metabolic risk.

These findings are consistent with previous evidence. Matsushita et al. reported that the CKD-EPI equation more accurately categorized risk for all-cause mortality and end-stage renal disease than the MDRD equation, reflecting improved prognostic stratification across a broad range of populations [9]. Although outcome-based performance was not assessed in the present study, this literature provides important context for interpreting the clinical relevance of equation-related classification differences in high-risk populations.

Important limitations of creatinine-based estimating equations should nevertheless be acknowledged, particularly in specific comorbidity groups. A meta-analysis by Lingli et al. showed that both MDRD and CKD-EPI tend to overestimate eGFR in individuals with diabetes, potentially delaying classification into early CKD stages, and that CKD-EPI may exhibit lower accuracy in diabetic populations compared with non-diabetic individuals, possibly due to factors such as hyperfiltration, obesity and chronic inflammatory states [22]. The heterogeneity observed across studies reinforces the need for cautious interpretation of eGFR values and supports the integration of complementary markers, such as albuminuria, to refine kidney risk stratification in these patients.

Heart failure represents a particularly illustrative high-risk subgroup. In this study, CKD-EPI classified a higher proportion of individuals with heart failure into CKD stages G3a–G5 across all phenotypes, with more pronounced differences in advanced stages. Among patients with heart failure and reduced ejection fraction, CKD-EPI classified 8.15% of individuals as stage G5 compared with 5.98% using MDRD, corresponding to an absolute difference of 2.17 percentage points and a relative increase of approximately 36%. Given the well-established association between CKD and heart failure, including increased hospitalization and mortality, such classification differences may be relevant when considering follow-up intensity, referral patterns and therapeutic decision-making, particularly regarding nephroprotective and cardioprotective therapies.

Overall, these results reinforce that higher CKD stage classification rates observed with CKD-EPI across comorbidity subgroups should be interpreted as equation-related classification effects rather than evidence of superior disease detection, underscoring the importance of a multidimensional approach to kidney risk assessment in primary healthcare.

### 4.3. Urinary Albumin-to-Creatinine Ratio

Albuminuria-stratified analyses revealed equation-related differences in eGFR-based CKD stage classification. In categories A1 (<30 mg/g) and A2 (30–300 mg/g), CKD-EPI classified a higher proportion of individuals into CKD stages, including G3a and G3b, compared with MDRD, with consistent relative increases across these categories. In contrast, differences between equations were less pronounced in category A3 (>300 mg/g), suggesting that when albuminuria is marked, kidney impairment is sufficiently advanced for both equations to yield similar stage classification, although CKD-EPI still tended to classify more individuals into advanced stages (G4 and G5).

When UACR data were unavailable, CKD-EPI also resulted in a higher proportion of individuals classified into CKD stages across all eGFR categories. This finding is particularly relevant in primary healthcare, where albuminuria assessment is frequently incomplete. Albuminuria represents a key marker of kidney damage and cardiovascular risk, and both reduced eGFR and albuminuria have been independently associated with mortality and progression to end-stage kidney disease [6,39].

These findings reinforce the importance of integrating eGFR and albuminuria in kidney risk assessment, particularly in multimorbidity contexts. As highlighted by Levin et al. and Sullivan et al., reliance on eGFR alone may lead to misclassification, especially among older adults, whereas the inclusion of albuminuria improves risk stratification and clinical interpretation [5,40].

Overall, although CKD-EPI yields higher CKD stage classification rates in this real-world primary care population, these results underscore the need for a multidimensional approach combining markers of kidney function and kidney damage. Given the high prevalence of CKD-associated conditions in this cohort, such integration is essential for effective CKD surveillance and management in primary healthcare settings [29].

### 4.4. Clinical Implications

The findings of this study have relevant practical implications for CKD surveillance in PHC. The choice of the eGFR-estimating equation substantially influences CKD stage classification, the risk profile of the monitored population, and subsequent clinical decision-making. In this large real-world cohort, CKD-EPI consistently resulted in higher CKD stage classification rates across the full spectrum of kidney function, particularly among individuals with high cardiovascular and metabolic risk. These classification differences may translate into earlier recognition of individuals at higher kidney and cardiovascular risk, with implications for follow-up intensity and care planning.

Continued use of the MDRD equation may contribute to a lower proportion of individuals being classified into CKD stages, not only in early stages but also in more advanced disease. In PHC settings—where screening, risk stratification, and longitudinal follow-up are central pillars of care—such classification differences may have implications for referral timing, monitoring strategies and therapeutic prioritization. From this perspective, the systematic use of CKD-EPI aligns with contemporary evidence and supports a more standardized approach to eGFR reporting in primary care.

Importantly, eGFR estimation should be interpreted in conjunction with markers of kidney damage, particularly albuminuria. The combined assessment of kidney function and kidney damage plays a central role in risk stratification and prognostic assessment, rather than diagnosis alone. Both reduced eGFR and albuminuria have been independently associated with adverse outcomes, including progression to end-stage kidney disease, cardiovascular events, and mortality. The results of the present study reinforce the importance of integrating eGFR and UACR to support a proactive and risk-oriented approach to CKD surveillance in PHC.

Equation choice also has implications for potential misclassification. Classification of individuals at low risk as having CKD may lead to unnecessary monitoring, treatment burden, patient anxiety, and avoidable healthcare costs, particularly in populations with low pre-test probability, such as younger adults. Conversely, underclassification of individuals at higher risk may delay intensified follow-up and contribute to progression toward more advanced CKD stages, with associated increases in morbidity, healthcare utilization, and costs [5,27]. These considerations highlight the need for population-adapted, cost-effective screening and stratification strategies.

At a system level, the high prevalence of CKD and its associated burden of adverse outcomes support the development of scalable, population-based interventions aimed at slowing disease progression and reducing cardiovascular risk—key objectives of any national kidney health plan. The findings of this study suggest that integrating automated eGFR calculation using CKD-EPI, alongside systematic assessment of albuminuria, may facilitate structured risk stratification and longitudinal monitoring, particularly among older adults.

Finally, given the successful application of this analysis within the Portuguese PHC system—characterized by proximity to patients, multidisciplinary teams, and longitudinal care—the organizational model underpinning these findings may offer transferable insights for CKD surveillance and management strategies in other healthcare systems.

### 4.5. Study Limitations

This retrospective study, based on electronic health records, has several limitations that should be acknowledged. As with all real-world data analyses, the quality and completeness of clinical records may constrain the interpretation of results. In particular, the absence of UACR data in a substantial proportion of individuals limited a comprehensive assessment of kidney risk and restricted albuminuria-stratified analyses.

In addition, kidney function assessment relied exclusively on serum creatinine–based estimating equations, without incorporation of cystatin C or direct measurement of glomerular filtration rate (GFR). This approach may have influenced eGFR estimation in specific subgroups, such as older adults and individuals with atypical body composition (e.g., severe obesity or sarcopenia). As highlighted by Delanaye et al., serum creatinine concentration is affected by non-renal determinants, including muscle mass, diet and tubular secretion, which may limit its individual reliability. In such contexts, combined equations using creatinine concentration and cystatin C, or direct GFR measurement with exogenous markers (e.g., iohexol), are more accurate and recommended strategies [12].

Importantly, CKD stage classification in this study was based on the most recent available serum creatinine concentration value for each individual, without confirmation of persistence over at least three months. As a result, the findings should be interpreted as reflecting differences in eGFR-based classification between equations, rather than confirmed CKD diagnosis, and some individuals classified into CKD stages may not meet formal criteria for chronicity. In addition, UACR data were unavailable in a relevant proportion of individuals, limiting complete KDIGO risk stratification. Approximately one quarter of these cases lacked albuminuria measurements, reflecting routine primary care practice and potentially leading to underestimation of kidney damage in some cases.

The analyzed data derive exclusively from individuals registered within the Matosinhos Health Unit, which may limit generalizability to other regions with different sociodemographic characteristics, organizational models or healthcare utilization patterns. According to national census data [41], the vast majority of residents in the study region hold Portuguese nationality (approximately 97%). However, nationality does not directly equate to ethnicity or genetic ancestry, and ethnicity is not systematically recorded in national electronic health records. Consequently, specific ethnic composition data were not available for analysis. This may limit the generalizability of the findings to more ethnically diverse populations, particularly given known variations in creatinine generation and eGFR estimation across different ancestry groups. Moreover, as the analysis was based on data generated through healthcare use, individuals who rarely access primary care services may be underrepresented, potentially influencing estimated CKD stage classification rates, particularly in earlier stages or less-monitored populations.

Furthermore, this study did not assess the direct clinical impact of equation choice on medical decision-making, follow-up strategies, or medium- and long-term outcomes, such as CKD progression, mortality or referral to nephrology. Future longitudinal studies linking equation-based classification differences to clinical outcomes are needed to address this gap.

Finally, the heterogeneity in kidney function assessment criteria, particularly in eGFR and albuminuria reporting, remains a challenge in both clinical care and research [28]. Variability in formula use and incomplete recording of key renal markers limit comparability across studies and hinder consistent application of international recommendations. These constraints underscore the need to standardize not only eGFR estimation methods but also the systematic recording of albuminuria in primary healthcare.

### 4.6. Strengths

An important strength of this study is the high degree of laboratory standardization. Unlike many multicenter analyses or studies based on large administrative databases, all serum creatinine concentration measurements were performed within the same laboratory network and by the same method. This methodological consistency minimizes inter-laboratory analytical variability, enhances comparability between estimating equations and strengthens the internal validity of the observed differences in eGFR-based CKD stage classification.

The importance of rigorous creatinine calibration for eGFR estimation has been widely emphasized in the literature, particularly in individuals with preserved kidney function, where small analytical variations may result in clinically relevant differences in CKD stage classification [42]. In this context, the analytical uniformity of the present study represents a substantial added value for the comparative evaluation of the CKD-EPI and MDRD equations.

Another major strength lies in the use of real-world data generated within PHC. The clinical information analyzed was recorded by primary care physicians during routine care, providing a highly representative view of patient trajectories in the community. This setting allows for the evaluation of eGFR-estimating equations as they are actually applied in daily clinical practice, capturing real patterns of disease burden, multimorbidity and risk stratification. The use of PHC data confers strong external validity to the findings and underscores the strategic role of primary care in generating epidemiologically robust and clinically meaningful evidence.

Despite the inherent limitations of observational real-world data, the large sample size, the high coverage of the regional adult population, and the exclusive focus on primary healthcare considerably enhance the robustness of the analyses and the practical relevance of the findings. Together, these strengths support the applicability of the results to clinical decision-making and health system planning in primary care-based settings.

### 4.7. Future Perspectives

This study opens the way for several lines of future research. A key priority will be prospective studies incorporating direct measurement of glomerular filtration rate and cystatin C assessment. Such approaches would enable more comprehensive validation of eGFR-estimating equations and a more precise evaluation of equation-related differences, particularly in specific clinical subgroups such as older adults, individuals with obesity or sarcopenia, and patients with heart failure.

In this context, further exploration of age-adapted interpretative frameworks for eGFR is warranted. Age-related physiological changes in kidney function may not be fully captured by current fixed cut-off points, and the use of age-dependent thresholds could help refine CKD stage classification in older populations while reducing misclassification related to physiological kidney aging.

Future research should also investigate the real-world impact of eGFR equation choice on clinical practice in primary healthcare, including CKD coding, referral patterns to nephrology, follow-up intensity and adherence to nephroprotective strategies. Linking equation-based classification differences to clinically relevant outcomes—such as hospitalization, cardiovascular events and mortality—will be essential to better understand their practical implications.

Additionally, the evaluation of combined stratification strategies integrating eGFR and albuminuria, as well as the development of automated, risk-based clinical decision support systems, may support more consistent identification and monitoring of individuals at higher risk of CKD progression.

Finally, the replication of this type of analysis in other regions and populations (distinct urban and rural contexts) is recommended to verify the consistency of observed patterns and adapt recommendations to local realities.

## 5. Conclusions

Chronic kidney disease represents a major global health challenge, with PHC playing a central role in population-level surveillance and risk assessment. In this large real-world PHC cohort, we demonstrated that the choice of eGFR-estimating equation substantially influences CKD stage classification.

Compared with MDRD, the CKD-EPI equation consistently resulted in a higher proportion of individuals classified into CKD stages G3–G5, corresponding to a relative increase of 17.6% in overall G3–G5 classification. These classification differences were particularly pronounced among older adults, across major cardiometabolic comorbidities, and across BMI categories. Differences were also observed in albuminuria-stratified analyses and in individuals without available UACR data, highlighting the impact of equation choice in routine primary care settings.

Importantly, these findings should be interpreted as differences in eGFR-based CKD stage classification, rather than as differences in confirmed CKD diagnosis. Nevertheless, such classification differences have relevant clinical and organizational implications, as they may influence risk stratification, follow-up intensity, referral patterns and healthcare resource planning in PHC.

Despite the retrospective design and reliance on routinely collected clinical data, the large sample size, high coverage of the regional adult population, and standardized laboratory methodology confer robustness and practical relevance to the results. This study contributes to a better understanding of how eGFR-estimating equations behave in real-world primary care and provides evidence to inform standardized equation selection in routine practice.

Future research should focus on prospective studies incorporating cystatin C and direct GFR assessment, as well as on linking equation-based classification differences to clinical outcomes. In the meantime, our findings provide real-world data relevant to ongoing efforts toward standardized CKD-EPI-based eGFR reporting in primary healthcare, within an integrated and risk-oriented framework for CKD surveillance and management.

## Figures and Tables

**Table 1:** GFR categories in CKD.

GFR Category	eGFR (mL/min/1.73 m^2^)
G1	≥90
G2	60–89
G3a	45–59
G3b	30–44
G4	15–29
G5	≤15

CKD, chronic kidney disease; GFR, glomerular filtration rate; eGFR, estimated glomerular filtration rate.

**Table 2:** Baseline demographic and anthropometric characteristics of the study population.

	*n*	%
Number of patients	117,055	100.00%
Male	49,709	42.47%
Female	67,346	57.53%
Age (years)		
18–30	14,854	12.69%
30–40	15,801	13.50%
40–50	19,882	16.99%
50–60	20,317	17.36%
60–70	20,073	17.15%
70–80	15,278	13.05%
≥80	10,850	9.27%
BMI ^1^ (kg/m^2^)		
<18.5	2888	2.47%
18.5–25.0	43,766	37.39%
25.0–30.0	42,768	36.54%
30.0–35.0	18,969	16.21%
35.0–40.0	5607	4.79%
≥40.0	2089	1.78%

^1^ BMI, body mass index.

**Table 3:** Clinical comorbidities of the study population.

	*n*	%
General Comorbidities		
Obesity	26,665	22.78%
Hypertension	55,697	47.58%
Hypercholesterolemia	53,540	45.74%
Type 2 Diabetes	30,161	25.77%
Structural Heart Disease	15,084	12.89%
Microvascular Disease	5242	4.48%
Cardiovascular Comorbidities		
Any CV ^1^ Disease	59,599	50.92%
Hypertension	55,697	47.58%
Atrial Fibrillation	5012	4.28%
CKD	9042	7.72%
Stable Angina	3622	3.09%
Transient Ischemic Attack	854	0.73%
Atherosclerotic Disease	10,736	9.17%
Unstable Angina	1768	1.51%
MI ^2^	2790	2.38%
Stroke	6563	5.61%
Ischemic Stroke	5362	4.58%
Stroke, Hemorrhagic	594	0.51%
Peripheral Artery Disease	3396	2.90%
Heart Failure		
Heart Failure Phenotype, Any	6148	5.25%
Preserved	2584	1.82%
Midrange	443	0.38%
Reduced	368	0.31%

^1^ CV disease, cardiovascular disease—defined as the presence of at least one of the cardiovascular conditions listed below; ^2^ MI, myocardial infarction.

**Table 4:** CKD stage classification by equation.

Stage	eGFR (mL/min/1.73 m^2^)	CKD-EPI ^1^		MDRD ^2^		Relative Difference
		*n*	%	*n*	%	%
GFR, latest		117,055	100.00%	117,055	100.00%	
G1	≥90	67,834	57.95%	63,527	54.27%	+6.8%
G2	60–89	40,179	34.32%	45,842	39.16%	−14.1%
G3a	45–59	5134	4.39%	4638	3.96%	+10.7%
G3b	30–44	2409	2.06%	1862	1.59%	+29.4%
G4	15–29	1089	0.93%	881	0.75%	+23.6%
G5	<15	410	0.35%	305	0.26%	+34.4%

^1^ CKD-EPI, Chronic Kidney Disease Epidemiology Collaboration; ^2^ MDRD, Modification of Diet in Renal Disease.

**Table 5:** CKD stage classification by estimating equation across age groups.

Age		[50, 60[		RD *	[60, 70[		RD	[70, 80[		RD	≥80		RD
		*n*	%	%	*n*	%	%	*n*	%	%	*n*	%	%
		20,317	17.36		20,073	17.15		15,278	13.05		10,850	9.27	
CKD-EPI	Stage, latest	20,317	100.00		20,073	100.00		15,278	100.00		10,850	100.00	
	Stage G3a	218	1.07	+16.6	834	4.15	−10.8	1694	11.09	+4.1	2298	21.18	+30.8
	Stage G3b	63	0.31	−15.9	274	1.37	+5.8	675	4.42	+34.2	1378	12.70	+36.8
	Stage G4	30	0.15	−10	108	0.54	+5.9	254	1.66	+17.1	671	6.18	+33.7
	Stage G5	28	0.14	+16.7	64	0.32	+25.5	96	0.63	+28	209	1.93	+47.2
MDRD	Stage, latest	20,317	100.00		20,073	100.00		15,278	100.00		10,850	100.00	
	Stage G3a	187	0.92		924	4.60		1628	10.66		1757	16.19	
	Stage G3b	73	0.36		259	1.29		503	3.29		1007	9.28	
	Stage G4	33	0.16		102	0.51		217	1.42		502	4.63	
	Stage G5	24	0.12		51	0.25		75	0.49		142	1.31	

* RD—Relative difference.

**Table 6:** CKD stage classification by estimating equation across BMI categories (<30 kg/m^2^).

BMI Brackets		<18.5		RD	[18.5, 25.0[		RD	[25.0, 30.0[		RD
		*n*	%	%	*n*	%	%	*n*	%	%
		2888	2.47		43,766	37.39		42,768	36.54	
CKD-EPI	Stage, latest	2888	100.00		43,766	100.00		42,768	100.00	
	Stage G3a	70	2.42	+228	1326	3.03	+17.3	2137	5.00	+12.6
	Stage G3b	51	1.77	+378	612	1.40	+29.9	961	2.25	+34.8
	Stage G4	22	0.76	+48	297	0.68	+21.7	402	0.94	+20.4
	Stage G5	14	0.48	+556	131	0.30	+43.9	156	0.36	+35.7
MDRD	Stage, latest	2888	100.00		43,766	100.00		42,768	100.00	
	Stage G3a	57	1.97		1130	2.58		1898	4.44	
	Stage G3b	37	1.28		471	1.08		713	1.67	
	Stage G4	21	0.73		244	0.56		334	0.78	
	Stage G5	9	0.31		91	0.21		115	0.27	

**Table 7:** CKD stage classification by estimating equation across BMI categories (≥30 kg/m^2^).

BMI Brackets		[30.0, 35.0[		RD	[35.0, 40.0[		RD	≥40.0		RD
		*n*	%		*n*	%		*n*	%	
		18,969	16.21		5607	4.79		2089	1.78	
CKD-EPI	Stage, latest	18,969	100.00		5607	100.00		2089	100.00	
	Stage G3a	1142	6.02	+5.1	313	5.58	−2.6	100	4.79	−7
	Stage G3b	521	2.75	+23.2	182	3.25	+20.5	58	2.78	+20.8
	Stage G4	244	1.29	+41.0	80	1.43	+17.6	30	1.44	+3.4
	Stage G5	70	0.37	+16.7	19	0.34	+35.7	14	0.67	+40
MDRD	Stage, latest	18,969	100.00		5607	100.00		2089	100.00	
	Stage G3a	1087	5.73		321	5.72		107	5.12	
	Stage G3b	423	2.23		151	2.69		48	2.30	
	Stage G4	173	0.91		68	1.21		29	1.39	
	Stage G5	60	0.32		14	0.25		10	0.48	

**Table 8:** CKD stage classification by estimating equation, stratified by comorbidity: Obesity, hypertension, hypercholesterolemia, type 2 diabetes and atherosclerotic disease.

		Obesity		Hypertension		Hypercholesterolemia		Type 2 Diabetes		Atherosclerotic Disease	
		*n*	%	*n*	%	*n*	%	*n*	%	*n*	%
		26,665	22.78	55,697	47.58	53,540	45.74	30,161	25.77	10,736	9.17
CKD-EPI	Stage, latest	26,665	100.00	55,697	100.00	53,540	100.00	30,161	100.00	10,736	100.00
	Stage G3a	1555	5.83	4684	8.41	1893	3.54	2794	9.26	1362	12.69
	Stage G3b	761	2.85	2274	4.08	751	1.40	1575	5.22	794	7.40
	Stage G4	354	1.33	1013	1.82	275	0.51	816	2.71	475	4.42
	Stage G5	103	0.39	374	0.67	132	0.25	296	0.98	204	1.90
MDRD	Stage, latest	26,665	100.00	55,697	100.00	53,540	100.00	30,161	100.00	10,736	100.00
	Stage G3a	1515	5.68	4201	7.54	1711	3.20	2632	8.73	1212	11.29
	Stage G3b	622	2.33	1749	3.14	583	1.09	1281	4.25	650	6.05
	Stage G4	270	1.01	818	1.47	219	0.41	664	2.20	408	3.80
	Stage G5	84	0.32	273	0.49	108	0.20	221	0.73	143	1.33

**Table 9:** CKD stage classification by estimating equation in patients with heart failure.

HF		Heart Failure, Any		Preserved		Midrange		Reduced	
		*n*	%	*n*	%	*n*	%	*n*	%
		6148	5.25	2135	1.82	443	0.38	368	0.31
CKD-EPI	Stage, latest	6148	100.00	2135	100.00	443	100.00	368	100.00
	Stage G3a	1040	16.92	374	17.52	75	16.93	62	16.85
	Stage G3b	870	14.15	302	14.15	71	16.03	57	15.49
	Stage G4	553	8.99	237	11.10	47	10.61	68	18.48
	Stage G5	258	4.20	126	5.90	26	5.87	30	8.15
MDRD	Stage, latest	6148	100.00	2135	100.00	443	100.00	368	100.00
	Stage G3a	1006	16.36	367	17.19	84	18.96	59	16.03
	Stage G3b	709	11.53	267	12.51	59	13.32	60	16.30
	Stage G4	459	7.47	191	8.95	41	9.26	62	16.85
	Stage G5	193	3.14	97	4.54	17	3.84	22	5.98

**Table 10:** CKD stage classification by eGFR stage and albuminuria category.

UACR * Staging		UACR A1 (<30)		UACR A2 (30–300)		UACR A3 (>300)		Chronic Kidney Disease UACR Unknown	
		*n*	%	*n*	%	*n*	%	*n*	%
		36,282	31.00	6470	5.53	1381	1.18	72,922	62.30
	Stage, latest	36,282	100.00	6470	100.00	1381	100.00	72,922	100.00
CKD-EPI	Stage G3a	2919	8.05	983	15.19	192	13.90	1040	1.43
	Stage G3b	1143	3.15	614	9.49	229	16.58	423	0.58
	Stage G4	376	1.04	311	4.81	223	16.15	179	0.25
	Stage G5	59	0.16	89	1.38	169	12.24	93	0.13
	Stage, latest	36,282	100.00	6470	100.00	1381	100.00	72,922	100.00
MDRD	Stage G3a	2511	6.92	949	14.67	207	14.99	971	1.33
	Stage G3b	842	2.32	503	7.77	212	15.35	305	0.42
	Stage G4	273	0.75	227	3.51	229	16.58	152	0.21
	Stage G5	39	0.11	67	1.04	127	9.20	72	0.10

* UACR: Urinary albumin-to-creatinine ratio (mg/g).

**Table 11:** CKD stage classification by eGFR stage and estimating equation among patients without albuminuria.

	Stage			CKD-EPI		UACR * Unknown		MDRD		UACR Unknown	
	GFR			*n*	%	*n*	%	*n*	%	*n*	%
GFR categories (mL/min/1.73 m^2^, description and range)	G3a	mildly to moderately decreased	45–59	5134	4.39	1040	20.3	4638	3.96	971	20.9
	G3b	moderately to severely decreased	30–44	2409	2.06	423	17.6	1862	1.59	305	16.4
	G4	severely decreased	15–29	1089	0.93	179	16.4	881	0.75	152	17.3
	G5	kidney failure	<15	410	0.35	93	22.7	305	0.26	72	23.6

* UACR: Urinary albumin-to-creatinine ratio.

## Data Availability

The datasets supporting the conclusions of this article are not publicly available due to data privacy restrictions concerning electronic health records. Access may be granted upon reasonable request to the corresponding author, subject to ethical approval and institutional data governance policies, as per the restrictions on availability outlined at the submission stage.

## Abbreviations

The following abbreviations are used in this manuscript:

BMI	Body Mass Index
BNP	B-type Natriuretic Peptide
CKD	Chronic Kidney Disease
CKD-EPI	CKD Epidemiology Collaboration
CV disease	Cardiovascular Disease
DBP	Diastolic Blood Pressure
eGFR	Estimated Glomerular Filtration Rate
GFR	Glomerular Filtration Rate
HbA1c	Hemoglobin A1c
HF	Heart Failure
HFmrEF	HF with Mildly Reduced Ejection Fraction
HFpEF	HF with Preserved Ejection Fraction
HFrEF	HF with Reduced Ejection Fraction
ICD-9/10	International Classification of Diseases, Ninth/Tenth Revision
ICPC-2	International Classification of Primary Care, Second Edition
KDIGO	Kidney Disease: Improving Global Outcomes
LVEF	Left Ventricular Ejection Fraction
MDRD	Modification of Diet in Renal Disease
MI	Myocardial Infarction
NT-proBNP	N-terminal pro-B-type Natriuretic Peptide
PHC	Primary Healthcare
RD	Relative Difference
SBP	Systolic Blood Pressure
SCr	Serum Creatinine Concentration
UACR	Urinary Albumin–Creatinine Ratio
ULSM	Unidade Local de Saúde de Matosinhos

## References

[1] Hill N.R., Fatoba S.T., Oke J.L., Hirst J.A., O’Callaghan C.A., Lasserson D.S., Hobbs F.D.R. (2016). Global Prevalence of Chronic Kidney Disease—A Systematic Review and Meta-Analysis. PLoS ONE.

[2] Bikbov B., Purcell C.A., Levey A.S., Smith M., Abdoli A., Abebe M., Adebayo O.M., Afarideh M., Agarwal S.K., Agudelo-Botero M. (2020). Global, Regional, and National Burden of Chronic Kidney Disease, 1990–2017: A Systematic Analysis for the Global Burden of Disease Study 2017. Lancet.

[3] McCullough K., Sharma P., Ali T., Khan I., Smith W.C.S., MacLeod A., Black C. (2012). Measuring the Population Burden of Chronic Kidney Disease: A Systematic Literature Review of the Estimated Prevalence of Impaired Kidney Function. Nephrol. Dial. Transplant..

[4] Jha V., Al-Ghamdi S.M.G., Li G., Wu M.-S., Stafylas P., Retat L., Card-Gowers J., Barone S., Cabrera C., Garcia Sanchez J.J. (2023). Global Economic Burden Associated with Chronic Kidney Disease: A Pragmatic Review of Medical Costs for the Inside CKD Research Programme. Adv. Ther..

[5] Levin A., Stevens P.E. (2011). Early Detection of CKD: The Benefits, Limitations and Effects on Prognosis. Nat. Rev. Nephrol..

[6] Coresh J., Turin T.C., Matsushita K., Sang Y., Ballew S.H., Appel L.J., Arima H., Chadban S.J., Cirillo M., Djurdjev O. (2014). Decline in Estimated Glomerular Filtration Rate and Subsequent Risk of End-Stage Renal Disease and Mortality. JAMA.

[7] Levey A.S., Bosch J.P., Lewis J.B., Greene T., Rogers N., Roth D. (1999). A More Accurate Method to Estimate Glomerular Filtration Rate from Serum Creatinine: A New Prediction Equation. Modification of Diet in Renal Disease Study Group. Ann. Intern. Med..

[8] Levey A., Stevens L., Schmid C., Zhang Y., Castro A.F., Feldman H., Kusek J., Eggers P., van Lente F., Greene T. (2009). A New Equation to Estimate Glomerular Filtration Rate. Ann. Intern. Med..

[9] Matsushita K., Mahmoodi B.K., Woodward M., Emberson J.R., Jafar T.H., Jee S.H., Polkinghorne K.R., Shankar A., Smith D.H., Tonelli M. (2012). Comparison of Risk Prediction Using the CKD-EPI Equation and the MDRD Study Equation for Estimated Glomerular Filtration Rate. JAMA.

[10] Ávila M., Mora Sánchez M.G., Bernal Amador A.S., Paniagua R. (2025). The Metabolism of Creatinine and Its Usefulness to Evaluate Kidney Function and Body Composition in Clinical Practice. Biomolecules.

[11] Kashani K., Rosner M.H., Ostermann M. (2020). Creatinine: From Physiology to Clinical Application. Eur. J. Intern. Med..

[12] Delanaye P., Pottel H., Cavalier E., Flamant M., Stehlé T., Mariat C. (2024). Diagnostic Standard: Assessing Glomerular Filtration Rate. Nephrol. Dial. Transplant..

[13] Levey A.S., Inker L.A., Coresh J. (2014). GFR Estimation: From Physiology to Public Health. Am. J. Kidney Dis..

[14] Froissart M., Rossert J., Jacquot C., Paillard M., Houillier P. (2005). Predictive Performance of the Modification of Diet in Renal Disease and Cockcroft-Gault Equations for Estimating Renal Function. J. Am. Soc. Nephrol..

[15] Michels W.M., Grootendorst D.C., Verduijn M., Elliott E.G., Dekker F.W., Krediet R.T. (2010). Performance of the Cockcroft-Gault, MDRD, and New CKD-EPI Formulas in Relation to GFR, Age, and Body Size. Clin. J. Am. Soc. Nephrol..

[16] van den Brand J.A.J.G., van Boekel G.A.J., Willems H.L., Kiemeney L.A.L.M., den Heijer M., Wetzels J.F.M. (2011). Introduction of the CKD-EPI Equation to Estimate Glomerular Filtration Rate in a Caucasian Population. Nephrol. Dial. Transplant..

[17] Inker L.A., Eneanya N.D., Coresh J., Tighiouart H., Wang D., Sang Y., Crews D.C., Doria A., Estrella M.M., Froissart M. (2021). New Creatinine- and Cystatin C-Based Equations to Estimate GFR without Race. N. Engl. J. Med..

[18] McFadden E.C., Hirst J.A., Verbakel J.Y., McLellan J.H., Hobbs F.D.R., Stevens R.J., O’Callaghan C.A., Lasserson D.S. (2018). Systematic Review and Metaanalysis Comparing the Bias and Accuracy of the Modification of Diet in Renal Disease and Chronic Kidney Disease Epidemiology Collaboration Equations in Community-Based Populations. Clin. Chem..

[19] Wang Y., Katzmarzyk P.T., Horswell R., Zhao W., Johnson J., Hu G. (2016). Comparison of the Heart Failure Risk Stratification Performance of the CKD-EPI Equation and the MDRD Equation for Estimated Glomerular Filtration Rate in Patients with Type 2 Diabetes. Diabet. Med..

[20] Delanaye P., Jager K.J., Bökenkamp A., Christensson A., Dubourg L., Eriksen B.O., Gaillard F., Gambaro G., van der Giet M., Glassock R.J. (2019). CKD: A Call for an Age-Adapted Definition. J. Am. Soc. Nephrol..

[21] Guebre-Egziabher F., Brunelle C., Thomas J., Pelletier C.C., Normand G., Juillard L., Dubourg L., Lemoine S. (2019). Estimated Glomerular Filtration Rate Bias in Participants with Severe Obesity regardless of Deindexation. Obesity.

[22] Lingli X., Qing Z., Wenfang X. (2020). Diagnostic Value of the Modification of Diet in Renal Disease and Chronic Kidney Disease Epidemiology Collaboration Equations in Diabetic Patients: A Systematic Review and Meta-Analysis. J. Int. Med. Res..

[23] Carter J.L., Stevens P.E., Irving J.E., Lamb E.J. (2011). Estimating Glomerular Filtration Rate: Comparison of the CKD-EPI and MDRD Equations in a Large UK Cohort with Particular Emphasis on the Effect of Age. QJM Int. J. Med..

[24] Ma Y., Shen X., Yong Z., Wei L., Zhao W. (2023). Comparison of Glomerular Filtration Rate Estimating Equations in Older Adults: A Systematic Review and Meta-Analysis. Arch. Gerontol. Geriatr..

[25] Elshahat S., Cockwell P., Maxwell A.P., Griffin M., O’Brien T., O’Neill C. (2020). The Impact of Chronic Kidney Disease on Developed Countries from a Health Economics Perspective: A Systematic Scoping Review. PLoS ONE.

[26] Sundström J., Bodegard J., Bollmann A., Vervloet M.G., Mark P.B., Karasik A., Taveira-Gomes T., Botana M., Birkeland K.I., Thuresson M. (2022). Prevalence, Outcomes, and Cost of Chronic Kidney Disease in a Contemporary Population of 2·4 Million Patients from 11 Countries: The CaReMe CKD Study. Lancet Reg. Health Eur..

[27] Sutton A.J., Breheny K., Deeks J., Khunti K., Sharpe C., Ottridge R.S., Stevens P.E., Cockwell P., Kalra P.A., Lamb E.J. (2015). Methods Used in Economic Evaluations of Chronic Kidney Disease Testing—A Systematic Review. PLoS ONE.

[28] Khan M.S., Bakris G.L., Packer M., Shahid I., Anker S.D., Fonarow G.C., Wanner C., Weir M.R., Zannad F., Butler J. (2022). Kidney Function Assessment and Endpoint Ascertainment in Clinical Trials. Eur. Heart J..

[29] Santos-Araújo C., Mendonça L., Carvalho D.S., Bernardo F., Pardal M., Couceiro J., Martinho H., Gavina C., Taveira-Gomes T., Dinis-Oliveira R.J. (2023). Twenty Years of Real-World Data to Estimate Chronic Kidney Disease Prevalence and Staging in an Unselected Population. Clin. Kidney J..

[30] Silva Junior G.B.D., Oliveira J.G.R.D., Oliveira M.R.B.D., Vieira L.J.E.D.S., Dias E.R. (2018). Global Costs Attributed to Chronic Kidney Disease: A Systematic Review. Rev. Assoc. Med. Bras..

[31] (2024). Kidney Disease: Improving Global Outcomes (KDIGO) CKD Work Group KDIGO 2024 Clinical Practice Guideline for the Evaluation and Management of Chronic Kidney Disease. Kidney Int..

[32] American Diabetes Association Professional Practice Committee (2024). 2. Diagnosis and Classification of Diabetes: Standards of Care in Diabetes-2024. Diabetes Care.

[33] McDonagh T.A., Metra M., Adamo M., Gardner R.S., Baumbach A., Böhm M., Burri H., Butler J., Čelutkienė J., Chioncel O. (2021). 2021 ESC Guidelines for the Diagnosis and Treatment of Acute and Chronic Heart Failure. Eur. Heart J..

[34] Brañez-Condorena A., Goicochea-Lugo S., Zafra-Tanaka J.H., Becerra-Chauca N., Failoc-Rojas V.E., Herrera-Añazco P., Taype-Rondan A. (2021). Performance of the CKD-EPI and MDRD Equations for Estimating Glomerular Filtration Rate: A Systematic Review of Latin American Studies. Sao Paulo Med. J..

[35] O’Callaghan C.A., Shine B., Lasserson D.S. (2011). Chronic Kidney Disease: A Large-Scale Population-Based Study of the Effects of Introducing the CKD-EPI Formula for eGFR Reporting. BMJ Open.

[36] Rocco M.V., Chapman A., Chertow G.M., Cohen D., Chen J., Cutler J.A., Diamond M.J., Freedman B.I., Hawfield A., Judd E. (2016). Chronic Kidney Disease Classification in Systolic Blood Pressure Intervention Trial: Comparison Using Modification of Diet in Renal Disease and CKD-Epidemiology Collaboration Definitions. Am. J. Nephrol..

[37] Garofalo C., Borrelli S., Minutolo R., Chiodini P., De Nicola L., Conte G. (2017). A Systematic Review and Meta-Analysis Suggests Obesity Predicts Onset of Chronic Kidney Disease in the General Population. Kidney Int..

[38] Pinto K.R.D., Feckinghaus C.M., Hirakata V.N. (2021). Obesity as a Predictive Factor for Chronic Kidney Disease in Adults: Systematic Review and Meta-Analysis. Braz. J. Med. Biol. Res..

[39] Hallan S.I., Matsushita K., Sang Y., Mahmoodi B.K., Black C., Ishani A., Kleefstra N., Naimark D., Roderick P., Tonelli M. (2012). Age and Association of Kidney Measures with Mortality and End-Stage Renal Disease. JAMA.

[40] Sullivan M.K., Rankin A.J., Jani B.D., Mair F.S., Mark P.B. (2020). Associations between Multimorbidity and Adverse Clinical Outcomes in Patients with Chronic Kidney Disease: A Systematic Review and Meta-Analysis. BMJ Open.

[41] Statistics Portugal—Web Portal. https://www.ine.pt/xurl/pub/66196836.

[42] Poggio E.D., Wang X., Greene T., Van Lente F., Hall P.M. (2005). Performance of the Modification of Diet in Renal Disease and Cockcroft-Gault Equations in the Estimation of GFR in Health and in Chronic Kidney Disease. J. Am. Soc. Nephrol..

